# Optimized Animal Models for the Genetic Evaluation of Conformation Traits, Milking Ease, and Milking Temperament in Dairy Gir Cattle

**DOI:** 10.3390/ani16030363

**Published:** 2026-01-23

**Authors:** Samla M. F. Cunha, Flavio S. Schenkel, Tatiane C. S. Chud, Anderson A. C. Alves, Marcos Vinícius G. B. da Silva, Rui da S. Verneque, João Cláudio do C. Panetto, Danísio P. Munari

**Affiliations:** 1 Departamento de Ciências Exatas, Faculdade de Ciências Agrárias e Veterinárias, Universidade Estadual Júlio de Mesquita Filho, Jaboticabal 14884-900, SP, Brazil; scunha@uoguelph.ca; 2 Centre for Genetic Improvement of Livestock, Department of Animal Biosciences, University of Guelph, Guelph, ON N1G 2W1, Canada; schenkel@uoguelph.ca (F.S.S.); tchud@uoguelph.ca (T.C.S.C.); 3 Department of Animal and Dairy Science, University of Georgia, Athens, GA 30602, USA; alvesand@uga.edu; 4 Embrapa Dairy Cattle, Juiz de Fora 36038-330, MG, Brazil; marcos.vb.silva@embrapa.br (M.V.G.B.d.S.); rui.verneque@embrapa.br (R.d.S.V.); joao.panetto@embrapa.br (J.C.d.C.P.)

**Keywords:** AIREML, categorical traits, genetic parameters, heritability, zebu cattle

## Abstract

This study aimed to evaluate four different models for the genetic evaluation of sixteen conformation traits, milking ease, and milking temperament in Dairy Gir cattle. The models vary based on whether they include only statistically significant fixed effects or all recorded effects, along with contemporary groups (CGs) treated as fixed or random effects. Categorical traits were also analyzed using a threshold model. The adjusted R-squared ($R_{adj}^{2}$) was used to compare the goodness-of-fit of the linear models. Spearman’s rank correlation and the average accuracy of bull estimated breeding values (EBVs) with at least 20 phenotyped daughters were compared. Models fitting CG as a random effect performed better based on their $R_{adj}^{2}$ values and had a greater average accuracy of EBVs for most traits. Spearman’s rank correlation coefficients indicated low to medium bull EBV re-ranking between most of the models. The linear models performed better than threshold models for almost all traits. When possible, more parsimonious linear models fitting only significant fixed effects should be used to reduce the standard error of estimation. Additionally, fitting CGs as a random effect seems more beneficial for the genetic evaluation of conformation and milking traits in Dairy Gir cattle in Brazil.

## Simple Summary

Enhancing the genetic evaluation of important traits can help increase productivity in dairy animals. This study focused on two groups of traits evaluated in Brazilian Dairy Gir cattle. Conformation traits, which describe the animal’s body and are linked to key production and health traits. Milking traits, which reflect the animals’ capacity to be milked and are especially important for zebu cattle, known for their responsiveness to the milking process. These traits are easy to measure, have moderate heritability, and are assessed early in the first lactation. The first step is to improve the statistical models used to estimate genetic parameters and breeding values. In this study, different models were tested to identify which provided the best goodness-of-fit, leading to the most suitable approach for evaluating conformation and milking traits. As a result, fitting only significant fixed effects in linear models and treating contemporary groups as random effects can be more advantageous for the genetic evaluation of conformation and milking traits in Dairy Gir cattle in Brazil. Implementing these findings in the genetic evaluation of the Dairy Gir breed can enhance genetic gains and ultimately improve farmers’ economic returns.

## 1. Introduction

In the Brazilian Dairy Gir National Breeding Program, eighteen linear traits are routinely evaluated using scores (from 1 to 9), centimeters, or angles. These traits include 16 body conformation traits, milking ease, and milking temperament. Conformation traits can be important indicators of herd life and general health [1,2]. Studies have shown that rump conformation traits impact calving ease, uterine and vaginal prolapse, and udder fixation [3,4]. Mammary system traits, mainly the anterior udder, udder depth, and teat length, are related to mastitis and susceptibility to trauma [5]. Traits such as milking ease and temperament, which evaluate animal behaviour during milking, are essential for effective daily dairy management [6]. Zebu breeds, such as Gir cattle, are generally more reactive than European breeds. This reactivity can lead to injuries for themselves and other animals, cause accidents, and prolong the milking process [7,8].

The estimation of breeding values and genetic parameters for these traits relies on optimized statistical models that explain the variability and produce reliable estimates. Some conformation and milking traits are categorical traits that are evaluated using scores. Hence, evaluating whether threshold models could fit these traits better than linear models is crucial. Theoretically, threshold models are preferable to linear models for categorical data [9], as linear models assume that residuals are normally distributed, which is typically not the case with score-based traits [10].

In dairy cattle, genetic evaluation models typically compare animal performance within groups, with the assumption that animals in the same group are raised in similar environments. These contemporary groups (CGs) can be defined by the combination of herd–year–season, herd–year, or their interactions [11]. Since 1973, when Henderson introduced the best linear unbiased prediction (BLUP) for the sire model evaluation [12], CGs have been treated as a fixed effect to avoid bias in situations where good sires are used in well-managed herds. However, this problem was mitigated with the implementation of animal models in evaluation systems, as CGs are genetically connected not only through sires but also through dams [13,14].

Given the importance of these traits in selecting productive and durable animals, understanding how poor statistical models can negatively influence genetic evaluations is crucial. Therefore, this study aimed to determine which models would perform better in the genetic evaluation of conformation traits, milking ease, and milking temperament in Dairy Gir cattle by testing the significance of fixed environmental effects to be included in the model and assessing the inclusion of CG as random effect in the model.

## 2. Materials and Methods

### 2.1. Data Description

The dataset used in this study was provided by Embrapa Dairy Cattle (Juiz de Fora, MG, Brazil), which is part of the Brazilian Dairy Gir National Breeding Program. The records were collected by the Brazilian Association of Dairy Gir Breeders and the Brazilian Association of Zebu Breeders between 1992 and 2018 from herds located in the southeastern region of Brazil.

The traits evaluated in this study included 16 conformation traits, milking ease, and milking temperament (Table 1). Stature (STA), heart girth (HG), body length (BL), navel length (NL), rump length (RL), pin width (PW), hook width (HW), teat length (TL), and teat diameter (TD) were measured in centimeters. Foot angle (FA), rear legs–side view (LSV), rear legs–rear view (LRV), fore udder attachment (FUA), rear udder width (RUW), udder depth (UD), milking ease (ME), and milking temperament (TEM) were recorded using a visual score (9-point scale). Rump angle (RA) was recorded in degrees using a slope inclinometer protractor or, in some cases, approximated from a visual linear score. Details on the traits were previously described by Panetto et al. [15].

The raw dataset contained 10,956 linear-type evaluations performed in the early stage of lactation on 7913 Gir cows. The number of evaluations per cow ranged from one to four, with 6153 cows being evaluated only once. The mean age of the cows at the evaluation was 1820 ± 788 days. Cows were born between 1976 and 2015 and calved between 1991 and 2018. The pedigree file used in the analysis for all traits consisted of 18,402 animals up to 15 generations.

### 2.2. Model Description

As the dataset included repeated measures, four alternative animal repeatability models were used (M1, M2, M3, and M4), as follows:

y=Xβ+Za+Wpe+e            (M1 and M2)

y=Xβ+Za+Wpe+Sd+e  (M3 and M4)

where y is the observation vector for the linear conformation traits, milking ease or milking temperament; X is the incidence matrix for fixed effects; Z is the incidence matrix for the animal additive genetic effects; W is the incidence matrix for the permanent environment effects of the animal; S is the incidence matrix for the CG effect; β is the vector of fixed environmental effects; a is the vector of random additive genetic effects; pe is the vector of random animal permanent environment effects; d is the vector of random CG (defined by animals in the same herd and year of evaluation) effects; and e is the vector of random residual effects. For models M1 and M2, CG was fit as a fixed effect in β.

For the models above, $Var\left(a\right)=A\sigma_{a}^{2}$, $Var\left(pe\right)=I\sigma_{pe}^{2}$, $Var\left(d\right)=I\sigma_{cg}^{2}$, and $Var\left(e\right)=I\sigma_{e}^{2}$ were assumed, where A is the additive relationship matrix; $\sigma_{a}^{2}$ is the additive genetic variance; I is an identity matrix; $\sigma_{pe}^{2}$ is the permanent environmental variance; $\sigma_{cg}^{2}$ is the contemporary group variance; and $\sigma_{e}^{2}$ is the residual variance. The genetic, permanent environment, contemporary group and residual effects were assumed to be independent.

Model M2 was the most complete model for all traits with respect to potential environmental effects, including all available environmental effects as fixed effects, except for the permanent environmental effect. Model M4 was similar to model M2, but with the CG effect treated as a random effect to exploit the use of the inter CG information and allow for the use of small-sized CG in the genetic evaluation. Model M1 was similar to model M2, but included just fixed environmental effects that were statistically significant to assess the use of more parsimonious models. Model M3 was similar to model M1, included only fixed environmental effects that were statistically significant to assess the use of more parsimonious models, but always included CG as a random effect. More details of the models are given below.

### 2.3. Models M2 and M4

The fixed effects considered in M2 and M4 included all recorded environmental effects, regardless of their statistical significance. For M2, CG (herd (H), year of evaluation (YE), and their interaction), season of evaluation (S), conformation trait evaluator (E), diet (D), and age of the dam as covariate (linear, L, and quadratic, Q, effects) were included as fixed effects. The random effects in M2 included animal and permanent environment effects. The fixed effects included in M4 were S, E, D, and the age of the dam as a covariate (with L and Q effects). The effects of the animal, the permanent environment, and the contemporary group were included as random effects.

For the eight traits measured with scores, the two models above were run as both linear models and threshold models. However, only linear models were fit for all other traits. See Section 2.5 for more details.

### 2.4. Models M1 and M3

A general linear model (GLM) was applied, using the R software [16] as a preliminary step to define which fixed effects would be included in models M1 and M3. Fixed effects were selected based on statistical significance (F test, *p* < 0.05) and biological relevance (Table 2). For M1, the fixed effects tested were H, YE, the interaction between H and YE, S, E, D, and the age of the dam as a covariate (L and Q effects). For M3, the same effects were tested, except for H, YE, and their interaction, which were fit as a random CG effect.

The random effects considered in M1 were animal and permanent environment effects. For M3, in addition to the CG, random animal and permanent environment effects were also included.

For eight traits measured with scores, the two models above were run as both linear models and threshold models. Therefore, only linear models were fit for all other traits. See Section 2.5 for more details.

### 2.5. Data Editing

Data that were outside of possible biological ranges for traits evaluated in cm and degrees were removed. Evaluators (between 13 and 23 evaluators were removed according to each trait) and CGs (between 542 and 570 CGs were removed according to each trait) with fewer than five records were removed from the analysis. The connectedness between CGs was assessed using the AMC program [17], in which the degree of connectedness is measured through the existence of genetic links attributed to a common ancestor. The program used ten or more genetic links to consider a CG connected, and any unconnected CG was removed (between 4 and 8 CGs were removed according to each trait). The descriptive statistics of this final dataset are provided in Table 1.

Due to differences in the models for BL, LSV, LRV, and TD, in which CG was not included either as a fixed effect or as a random effect in M1, because the CG effect was not significant for these traits, a second dataset was created, which was not edited based on CG size and connectedness. Disconnected CGs or those with fewer than five records within each group were not removed for these traits in M1 (Table 3). This dataset (dataset 2) was used exclusively for the analysis of the traits BL, LSV, LRV, and TD under M1. The remaining trait for M1 and all traits under M2, M3, and M4 were evaluated using dataset 1. Despite the differences in the number of animals used in both datasets, the means and standard deviations were similar across the datasets (Table 1 and Table 3).

### 2.6. Data Distribution and Transformation

Skewness and kurtosis tests were performed in R software (version 4.5.2) [16] using the R package “moments” (version 0.14.1) [18] to verify distribution properties of the categorical traits. Several data transformations were tested when substantial deviations from symmetry were observed, including log transformation, square root transformation, cube root transformation, Yeo–Johnson transformation, and ordered quantile normalization transformation. These transformations were performed using base R or the “fitdistrplus” (version 1.2-4) [19] and “bestNormalize” (version 1.9.2) [20] packages. The ordered quantile normalization method [20] was chosen to transform the score records because it yielded the best skewness and kurtosis values after the transformation. The transformed data were used for the analyses of the linear models. For the threshold models, the original data were used for the analyses.

### 2.7. Genetic Parameter Estimates

Variance components were estimated using the average information restricted maximum likelihood (AIREML) method [21] under a single-trait repeatability animal linear model and threshold model. All analyses were conducted using ASREML 4.1 software [22].

For the multinomial threshold analyses, the !MULTINOMIAL qualifier and the !LOGIT function were used in ASREML 4.1 software. The logit link function is as follows:

$$\eta =\log\left(\frac{µ}{1-µ}\right)$$

where µ is the mean on the data scale and η is the linear predictor on the underlying scale. On the logit scale, the residual variance is as follows:

$$\sigma_{e}^{2}=\left(\frac{\pi^{2}}{3}\right)\approx 3.29$$

Heritability ($h^{2}$) and repeatability (r) estimates for the threshold models were estimated as follows:

$$h^{2}=\frac{\sigma_{a}^{2}}{\sigma_{a}^{2}+\sigma_{pe}^{2}+\frac{\pi^{2}}{3}}\;\;and\;r=\frac{\sigma_{a}^{2}+\sigma_{pe}^{2}}{\sigma_{a}^{2}+\sigma_{pe}^{2}+\frac{\pi^{2}}{3}}\;$$

where $\sigma_{a}^{2}$ is the additive genetic variance and $\sigma_{pe}^{2}$ is the permanent environmental variance [22]. The heritability ($h^{2}$) and repeatability (r) of the linear models were calculated as follows:

$$h^{2}=\frac{\sigma_{a}^{2}}{\sigma_{a}^{2}+\sigma_{pe}^{2}+\sigma_{e}^{2}}\;\;and\;r=\frac{\sigma_{a}^{2}+\sigma_{pe}^{2}}{\sigma_{a}^{2}+\sigma_{pe}^{2}+\sigma_{e}^{2}}$$

where $\sigma_{e}^{2}$ is the residual variance.

For models that include CG as a random effect (M3 and M4), the denominator of the $h^{2}$ and r equations also include the CG variance ($\sigma_{cg}^{2}$).

### 2.8. Model Evaluation Criteria

First, models with all fixed effects included were compared to models that included only significant effects (M1 versus M2). Then, the inclusion of CG as fixed or random effects was compared (M1 versus M3 or M2 versus M4). In the first comparison, the structure of the fixed effects changes between models, and in the second, the structure of both the fixed and random effects changes at the same time, making the use of an appropriate parameter to compare the models difficult. As noted by Verbyla [23], models that vary in the structure of their fixed effects cannot have their restricted (residual) likelihood used to compare the models. Instead, the full and maximum likelihoods should be used. Because ASREML 4.1 software uses and outputs the restricted maximum likelihood rather than the maximum likelihood of the models, in this study, we chose to use the adjusted R-squared ($R_{adj}^{2}$) value, which is a measurement that adjusts the goodness-of-fit to the number of fixed effects considered in the model. $R_{adj}^{2}$ was calculated for each model and trait individually as follows:

$$R_{adj}^{2}=1-\frac{\left(N-1)(1-R^{2}\right)}{\left(N-r\left(x\right)\right)}$$

where N is the number of records; $R_{adj}^{2}$ is the correlation between y and $\hat{y}$ squared, calculated as $R^{2}=\left[{cor(y,\hat{y})}^{2}\right]$; and *r*(x) is the sum of the degrees of freedom. A higher value of $R_{adj}^{2}$ indicates that the model explains more of the phenotype. Even thought, $R_{adj}^{2}$ is not the best criterion to compare mixed models, as it does not take into account the partition of the variance across random effects, it would be a better alternative than using restricted likelihood based criteria in the case of alternative mixed models that vary in their fixed effects.

### 2.9. Impacts of the Models on the Animal Evaluation

The average accuracy of the estimated breeding value (EBV) of bulls with at least 20 phenotyped daughters for each trait was calculated for all the models. The accuracy of the EBV for each bull was estimated as follows:

$${acc}_{i}=\sqrt{\left(1-\frac{{SE}_{i}^{2}}{\left(1+f_{i}\right)\sigma_{a}^{2}}\right)}$$

where ${SE}_{i}$ is the standard error of prediction of the EBV of the ith animal as estimated by ASREML; $f_{i}$ is the inbreeding coefficient of the ith animal; and $\sigma_{a}^{2}$ is the population additive genetic variance [24]. A paired Student’s *t*-test was used to test differences (*p* < 0.05) between the average EBV accuracy of bulls with at least 20 phenotyped daughters between the alternative models for each trait.

Additionally, Spearman’s rank correlation coefficient was computed between the EBV for the same bulls used to calculate the average EBV accuracies to assess the impact of using different models in the ranking of the bulls used as parents in the next generations. The comparisons were based on linear model M1 versus M2, M1 versus M3, M2 versus M4, linear model M3 versus threshold model M3, and linear model M4 versus threshold model M4.

## 3. Results and Discussion

Optimizing models to be used in the genetic evaluation of important economic traits is necessary since they can improve the estimation of genetic parameters, breeding values, and accuracies of predictions. An optimized model can impact the selection of animals to be the sires and dams of the next generations and, consequently, can lead to greater genetic gain and increased profitability. This study compared different models for the genetic evaluation of conformation traits, milking ease, and milking temperament in Dairy Gir cattle to identify the most suitable model. The models differed in their fixed effect structure and the inclusion of contemporary groups as fixed or random effects. Additionally, linear and multinomial threshold models were compared for the genetic evaluation of the categorical traits.

The results and discussion that follow present genetic parameter estimates across all traits and models, with a comparison of goodness of fit, average EBV accuracies, and bulls’ EBV rankings of linear models for groups of traits, followed by a comparison between linear and threshold models for categorical traits, and a summary of results and limitations of this study.

### 3.1. Genetic Parameter Estimates

The estimates of additive genetic, permanent environmental, and residual variances for all the evaluated models are shown in Table 4. Heritability and repeatability estimates are presented in Table 5 for both linear and threshold models. The estimated standard errors for the variance, heritability and repeatability estimates are presented in Table S1 and Table S2 (Supplementary Material), respectively.

The estimates of genetic, permanent environmental, and residual variances were similar between the linear models for almost all the traits (Table 4). Body length, rear legs–side view, rear legs–rear view, and teat diameter had a smaller estimated residual variance for linear models M2, M3, and M4 than for linear model M1. This result may be because for these traits, CG was not included in the linear model M1 but it was included in the linear models M2, M3, and M4 as a fixed or random effect, which could have reduce the residual variance.

The estimates of additive genetic variance for teat diameter and body length were higher for linear model M1 than for M2, M3, and M4. However, estimates of permanent environmental variance were higher for linear models M2, M3, and M4 than for linear model M1. The linear M2, M3, and M4 models included CG (herd–year evaluation) for both traits, either as a fixed or random effect, whereas linear model M1 included only the year of evaluation. The difference in the genetic additive variance is reflected in a higher heritability estimate for linear model M1 for these traits. However, the repeatability estimates were similar between linear models M1 and M2 (Table 5).

Small differences in the repeatability estimates for the hook width between linear models M1 and M2 and linear models M3 and M4 were observed, which could be attributed to the inclusion of the evaluator as a fixed effect in M2 and M4.

The estimates of genetic additive and permanent environment variance were very similar for the threshold models M1 and M2 for all the traits, except for the rear legs–side view, in which M1 showed smaller estimates of genetic additive and permanent environment variance (0.47 and 1.68, respectively) than did M2 (0.71 and 2.50, respectively) (Table 4). These differences may be attributed to the fixed effects (CG and quadratic effect of the dam’s age) added to the second model. When comparing threshold models M1 versus M3 and M2 versus M4, the estimates of genetic additive and permanent environment variance were smaller for M3 and M4 for almost all traits, except for rear legs–side view and rear udder width, which is expected since one more random effect (CG) was included in threshold models M3 and M4.

The estimates of genetic additive variance (Table 4) and heritability (Table 5) for rear legs–rear view were low and not reliable for all four models when both linear and threshold models were used, which may be due to the inability to record this trait correctly in the field or the poor definition of the trait. Traits related to feet and legs are highly influenced by the environment and management [25,26], which explains the low heritability estimates. Heritability estimates in the literature for rear legs–rear view are scarce; however, Duru et al. [27] reported a heritability of 0.11 (±0.18), and Ptak et al. [28] reported a heritability equal to 0.09 (±0.02) for this trait.

When threshold models M1 and M2 were compared, heritability and repeatability estimates were similar between models (Table 5). As expected, thresholds M3 and M4, which included CG as a random effect, yielded estimates of heritability and repeatability smaller than those of M1 and M2, which included CG as a fixed effect (Table 5). This result is expected because the inclusion of CG variance in the estimated phenotypic variance leads to a higher denominator in the fraction, resulting in a smaller heritability and repeatability estimate, which should be interpreted as estimates across CGs in contrast to the estimates within CG, which are obtained when CG is treated as a fixed effect and, therefore, does not contribute to the estimated phenotypic variance.

Heritability estimates for conformation traits, milking ease, and milking temperament vary in the literature, which could be due to several factors, including the statistical model, breed studied, fixed and random effects considered in the models, and data editing, among others [29]. However, the heritability estimates in this study were generally similar to the results in the literature [27,30,31,32,33,34,35,36].

### 3.2. Heart Girth, Rump Length, Teat Length, and Navel Length

For heart girth, rump length, teat length, and navel length, all tested fixed effects were significant (*p* < 0.05), resulting in the same models for M1 and M2 (Table 2) and the same values of $R_{adj}^{2}$. Consequently, M3 and M4 were also the same models with the same $R_{adj}^{2}$ values. When the linear models with CGs fitted as a fixed effect (M1 and M2) were compared with the linear models with CG fitted as a random effect (M3 and M4), the linear models M3 and M4 showed a better fit based on their higher $R_{adj}^{2}$ values (Table 6). For these traits, the average EBV accuracies were the same between M3 and M4 (Table 7). The differences in average EBV accuracy of bulls with at least 20 daughters with records between M1 and M3 or M2 and M4 were statistically significant (*p* < 0.05) for all compared traits (Table S3 in the Supplementary Material). The EBV Spearman rank correlation coefficients between M1 and M3 (or M2 and M4) for these traits ranged between 0.98 and 0.99 (Table 8), which indicates that changing the CG from fixed to random effect had little impact on the ranking of bulls for these traits.

### 3.3. Stature, Pin Width, Rump Angle, Udder Depth, Milking Ease, and Milking Temperament

Stature, pin width, rump angle, udder depth, milking ease, and milking temperament showed the same $R_{adj}^{2}$ values between linear models M1 and M2 (Table 6), which could indicate that differences in the structure of fixed effects for these traits were not sufficient to affect their $R_{adj}^{2}$ values. The $R_{adj}^{2}$ values between linear models M3 and M4 for these traits were the same. However, when the linear models M1 and M2 were compared with M3 and M4, respectively, the models that included CG as a random effect showed higher values of $R_{adj}^{2}$, indicating a better fit of the linear models M3 and M4 with CG as a random effect. Although the $R_{adj}^{2}$ values were the same between M3 and M4, fitting nonsignificant fixed effects in a model can lead to increased standard error of the estimates. When the average EBV accuracies for these traits were compared between linear models M1 versus M3 and M2 versus M4, linear M3 and M4 presented higher averages for all traits, except udder depth and milking temperament, which presented similar values (Table 7). The differences in average EBV accuracy for bulls with at least 20 daughters with records between M1 and M3 and between M2 and M4 were statistically significant (*p* < 0.05) for all compared traits, except for udder depth (Table S3 in the Supplementary Material). Similarly, the EBV Spearman rank correlation coefficient between linear M1 and M2 was greater than 0.99 (Table 8) for these traits, indicating that differences in the fixed effects fitted in the model led to little or no reranking between both models, also supporting the use of more parsimonious models. The EBV Spearman rank correlation coefficients between linear models M1 and M3 and between M2 and M4 were similar and ranged from 0.97 to 0.99 (Table 8), indicating that just a few bulls were reranked, especially for pin width and udder depth, when CG was considered a random effect.

### 3.4. Rear Legs–Side and –Rear Views

For the rear legs–side and –rear views, linear model M1 showed higher values of $R_{adj}^{2}$ than the linear model M2. When linear model M1 was compared to linear model M3, linear model M3 showed higher values of $R_{adj}^{2}$ (Table 6). This result could indicate that considering CG as a random effect could have led to better model performance for the rear legs–side and –rear views. The average EBV accuracies for these traits were greater for linear model M1 (rear legs–side view) and M4 (rear legs–rear view) (Table 7). A higher average EBV accuracy for linear model M1 for the rear legs–side view could be explained by a slightly higher additive genetic variance estimated for this model. Differences in average EBV accuracy for bulls with at least 20 daughters with records were statistically significant (*p* < 0.05) for rear legs–side and –rear views between M1 and M2, M1 and M3, and M2 and M4 (Table S3 in the Supplementary Material). The EBV Spearman rank correlation coefficient between linear models M1 and M2 indicated greater reranking when the fixed effects changed between models for rear legs–side view (0.91) than for rear legs–rear view (0.68). The removal of CG from the models, due to its lack of significance, impacted the bulls’ rankings, as well as the genetic parameter estimates, as discussed previously. A large reranking was also observed when linear models M1 and M3 were compared for rear legs–rear view (0.80, Table 8) and again a much smaller re-ranking was found for rear legs–side view (0.94). Changing the CG from fixed to random effect impacted much less the ranking (0.95 and 0.99) when comparing linear models M2 and M4 for rear legs–rear and –side views, respectively.

### 3.5. Body Length, Hook Width, Foot Angle, Fore Udder Attachment, Rear Udder Width, and Teat Diameter

The body length, hook width, foot angle, fore udder attachment, rear udder width, and teat diameter showed higher values of $R_{adj}^{2}$ (Table 6) for linear model M2 than for linear model M1. When the linear model M2 was compared to the linear model M4, the linear model M4 presented higher values of $R_{adj}^{2}$, indicating a better fit of this model for these traits. The average EBV accuracies were higher for linear model M4 for foot angle and hook width. For body length and teat diameter, linear model M1 had the highest average EBV accuracies, and for fore udder attachment and rear udder width, linear model M2 showed the highest average EBV accuracies (Table 7). The highest average EBV accuracies found for linear models M1 and M2 for body length and teat diameter and for fore udder attachment and rear udder width could be explained by higher additive genetic variance estimated for these models and traits, respectively. Differences in average EBV accuracy for bulls with at least 20 daughters with records were statistically significant (*p* < 0.05) for all compared traits between M1 and M2, M1 and M3, and M2 and M4 (Table S3 in the Supplementary Material). Removing CG as an effect from linear model M1 for body length and teat diameter may explain the moderately high reranking observed between linear models M1 and M2 (0.88 and 0.89) and linear models M1 and M3 (0.92) (Table 8). Little reranking was observed between linear models M2 and M4 (0.98 to 0.95) for body length, hook width, foot angle, fore udder attachment, rear udder width, and teat diameter.

### 3.6. Linear and Threshold Models

A comparison of the $R_{adj}^{2}$ values between the linear and threshold models is not possible since they are in different scales. However, an evaluation of the impacts of both models on average EBV accuracies and the bull rankings is possible. When comparing the linear model with higher $R_{adj}^{2}$ values (M3: rear legs–side and –rear views; or M4: foot angle, fore udder attachment, rear udder width, udder depth, milking ease, and milking temperament) to their respective thresholds for the categorical traits, the average EBV accuracies were higher for the linear models (M3 and M4) than for the threshold models (M3 and M4), except for milking ease (Table 7). The differences in average EBV accuracy for bulls with at least 20 daughters with records between the linear and threshold models were statistically significant (*p* < 0.05) for all compared traits, except for milking temperament for linear model M3 versus threshold model M3 and milking ease for linear model M4 versus threshold model M4 (Table S3 in the Supplementary Material). The EBV Spearman rank correlation coefficients were greater than 0.94 for all traits (Table 8) indicating a low degree of reranking between linear and threshold models. The similar results found between linear and threshold models can also be attributed to the transformation of the categorical data to fit normality when linear models were applied. The use of non-normal distributed data can lead to biased genetic parameter estimates and improper animal ranking.

Meijering [37] reported no advantage of using threshold models over linear models, although threshold models were theoretically a better option. Vanderick et al. [38] observed that the threshold model had better goodness of fit than linear models. However, according to the authors, no clear advantage was found in terms of predictive ability, and linear models would be more suitable and practical for application in genetic evaluation. Weller et al. [39] also identified several advantages of using threshold models over linear models, and even though threshold models estimate larger variance components, the rank correlation estimated between the two models was greater than 0.90. Threshold models can theoretically be the best fit for categorical traits. However, this study revealed that the lower average EBV accuracies and minimal changes in the animals’ rankings for most of the studied traits may not justify the implementation of these models in a genetic evaluation.

### 3.7. Summary and Study Limitations

In this study, models including all fixed effects (M2) performed better or the same as models that included only statistically significant fixed effects (M1) based on their $R_{adj}^{2}$ values for most traits, except rear legs–side and –rear views. The inclusion or exclusion of fixed effects in a model needs to be performed carefully. The inclusion of nonsignificant fixed effects can lead to overparameterization of the statistical models and increase the standard error of the estimates. However, when important effects are not included in the model can lead to biases in the evaluation. For traits with the same $R_{adj}^{2}$ values between linear models M1 and M2, using a model that includes only significant fixed effects may be recommended to reduce the standard error of the estimates.

Models incorporating CG as a random effect showed better fitting across all assessed traits in this study, as indicated by their higher $R_{adj}^{2}$ values. As suggested by Schaeffer [13], when an animal model is used, CG should be treated as a random effect, whereas CG was commonly treated as a fixed effect in the past based on Henderson’s selection bias theory under a sire model. Fitting CG as a random effect can result in other benefits. For instance, when CG is treated as a random effect, the loss of information from the removal of CGs with a small number of individuals (e.g., less than 5) would be avoided [40].

A limitation of this study is the use of $R_{adj}^{2}\;$ to compare linear mixed models. This limitation could be resolved if the models’ maximum likelihood values, instead of restricted maximum likelihood values, were available, which could be used to compare models that differ in their fixed effects.

## 4. Conclusions

This study provides valuable information for the optimization of models for future applications in the genetic evaluation of conformation traits, milking ease, and milking temperament in Dairy Gir cattle in Brazil. When linear and threshold models were compared, no advantages were observed for the threshold model. The inclusion of only significant environmental fixed effects in the model should be considered instead of all recorded environmental effects for some traits to avoid overparameterization and increased standard errors of estimation. Finally, contemporary group should be included as a random effect in the animal models since this modelling provided better model fitting and higher EBV accuracies and little bull EBV re-ranking.

## Figures and Tables

**Table 1:** Descriptive statistics for all traits evaluated using dataset 1.

Trait	N	Min	Max	Mean	SD	Median
Stature (cm)	9677	119	155	136.90	4.92	137
Hearth girth (cm)	9529	141	210	175.90	8.91	175
Body length (cm)	9387	86	127	103.50	5.98	103
Rump length (cm)	9652	31	59	42.76	4.30	43
Pin width (cm)	9520	10	29	18.25	2.62	18
Hook width (cm)	9501	33	62	46.27	4.37	47
Rump angle (degree)	5936	1	50	24.42	7.73	25
Foot angle (score)	8746	1	9	-	-	5
Rear legs–side view (score)	6035	1	9	-	-	5
Rear legs–rear view (score)	6029	1	9	-	-	5
Fore udder attachment (score)	5617	1	9	-	-	5
Rear udder width (score)	5822	1	9	-	-	5
Udder depth (score)	6003	1	9	-	-	5
Teat length (cm)	9690	3	14	7.51	1.92	7
Teat diameter (cm)	9666	1	8	3.87	0.87	4
Navel length (cm)	5539	1	20	10.20	3.16	10
Milking ease (score)	9486	1	9	-	-	3
Milking temperament (score)	9672	1	9	-	-	4

N: number of records; Min: minimum value for the evaluated trait; Max: maximum value for the evaluated trait; Mean: mean; SD: standard deviation from the mean; Median: median by trait. -: do not apply.

**Table 2:** Fixed effects included in the statistical models 1 (M1) and 3 (M3) used for the genetic evaluation of linear conformation traits, milking ease, and milking temperament in Dairy Gir cattle.

Trait	M1	M3
Stature	CG + E + D + L + Q	E + D + L + Q
Hearth Girth	CG + S + E + D + L+ Q	S + E + D + L + Q
Body length	YE + S + E + D + L + Q	S + E + D + L + Q
Rump length	CG + S + E + D + L + Q	S + E + D + L + Q
Pin width	CG + S + E + L + Q	S + E + L + Q
Hook width	CG + D + L + Q	D + L + Q
Rump angle	CG + S + E + D + L	S + E + D + L + Q
Foot angle	CG + S + D + L + Q	S + D + L + Q
Rear legs–side view	S + E + D + L	S + E + D + L
Rear legs–rear view	E + D	E + D
Fore udder attachment	CG + S + D + L	S + D + L
Rear udder width	CG + S + D + L	S + D + L
Udder depth	CG + E + D + L + Q	E + D + L + Q
Teat length	CG + S + E + D + L + Q	S + E + D + L + Q
Teat diameter	YE + E + L + Q	E + L + Q
Navel length	CG + S + E + D + L + Q	S + E + D + L + Q
Milking ease	CG + E + L + Q	E + L + Q
Milking temperament	CG + S + E + L + Q	S + E + L + Q

CG: contemporary group (herd + year of evaluation); YE: year of evaluation; S: season of evaluation; E: evaluator; D: diet effect; L: linear effect of age of the dam; Q: quadratic effect of age of the dam.

**Table 3:** Descriptive statistics for all traits evaluated using dataset 2.

Trait	N	Min	Max	Mean	SD	Median
Body length (cm)	10,504	86	127	103.50	5.95	103
Rear legs–side view (score)	7200	1	9	-	-	5
Rear legs–rear view (score)	7194	1	9	-	-	5
Teat diameter (cm)	10,802	1	8	3.89	0.91	4

N: number of records; Min: minimum value for the evaluated trait; Max: maximum value for the evaluated trait; Mean: mean; SD: standard deviation from the mean; Median: median by trait. -: do not apply.

**Table 4:** Additive genetic ($\sigma_{a}^{2}$), permanent environmental ($\sigma_{pe}^{2}$), contemporary group ($\sigma_{cg}^{2}$), and residual variances ($\sigma_{e}^{2}$) estimated for all evaluated traits using linear and threshold models.

Traits	M1 †			M2 †			M3 †				M4 †				M1 ‡§		M2 ‡§		M3 ‡§			M4 ‡§		
	$\sigma_{a}^{2}$	$\sigma_{pe}^{2}$	$\sigma_{e}^{2}$	$\sigma_{a}^{2}$	$\sigma_{pe}^{2}$	$\sigma_{e}^{2}$	$\sigma_{a}^{2}$	$\sigma_{pe}^{2}$	$\sigma_{cg}^{2}$	$\sigma_{e}^{2}$	$\sigma_{a}^{2}$	$\sigma_{pe}^{2}$	$\sigma_{cg}^{2}$	$\sigma_{e}^{2}$	$\sigma_{a}^{2}$	$\sigma_{pe}^{2}$	$\sigma_{a}^{2}$	$\sigma_{pe}^{2}$	$\sigma_{a}^{2}$	$\sigma_{pe}^{2}$	$\sigma_{cg}^{2}$	$\sigma_{a}^{2}$	$\sigma_{pe}^{2}$	$\sigma_{cg}^{2}$
STA	8.54	5.00	3.54	8.54	5.00	3.53	8.90	4.87	3.29	3.54	8.90	4.87	3.28	3.54	-	-	-	-	-	-	-	-	-	-
HG ^a^	12.44	18.53	17.49	12.44	18.53	17.49	14.35	17.39	21.42	17.54	14.35	17.39	21.42	17.54	-	-	-	-	-	-	-	-	-	-
BL	7.65	2.74	15.76	4.46	4.12	14.18	5.00	3.67	4.73	14.27	5.00	3.67	4.73	14.27	-	-	-	-	-	-	-	-	-	-
RL ^a^	1.16	1.55	2.53	1.16	1.55	2.53	1.32	1.40	2.52	2.62	1.32	1.40	2.52	2.62	-	-	-	-	-	-	-	-	-	-
PW	0.65	0.67	1.77	0.65	0.66	1.77	0.66	0.63	2.19	1.82	0.66	0.63	2.15	1.82	-	-	-	-	-	-	-	-	-	-
HW	1.33	1.35	5.01	1.37	1.95	4.06	1.39	1.32	9.24	5.00	1.55	1.82	4.04	4.11	-	-	-	-	-	-	-	-	-	-
RA	3.54	8.76	17.66	3.54	8.76	17.66	3.43	8.70	7.32	17.86	3.43	8.71	7.32	17.86	-	-	-	-	-	-	-	-	-	-
FA	0.05	0.10	0.56	0.05	0.11	0.53	0.05	0.09	0.19	0.56	0.05	0.10	0.10	0.55	0.31	1.16	0.33	1.29	0.28	0.60	1.18	0.29	0.66	0.67
LSV	0.07	0.22	0.50	0.07	0.20	0.45	0.06	0.20	0.07	0.45	0.06	0.20	0.07	0.45	0.47	1.68	0.71	2.50	0.49	1.53	0.52	0.49	1.53	0.52
LRV	0.001	0.27	0.47	0.004	0.26	0.42	0.005	0.26	0.05	0.42	0.006	0.26	0.05	0.42	0.00	1.76	0.04	2.51	0.03	1.65	0.40	0.03	1.66	0.40
FUA	0.04	0.32	0.29	0.05	0.31	0.28	0.04	0.32	0.27	0.29	0.04	0.31	0.08	0.29	0.54	5.70	0.64	5.74	0.36	3.31	2.74	0.41	3.62	0.87
RUW	0.03	0.14	0.29	0.04	0.13	0.28	0.03	0.15	0.47	0.29	0.04	0.14	0.13	0.28	0.50	3.09	0.58	3.18	0.92	0.00	5.73	0.38	1.69	2.01
UD	0.08	0.16	0.44	0.08	0.16	0.44	0.07	0.16	0.10	0.44	0.07	0.16	0.10	0.44	0.82	2.99	0.83	2.99	0.58	1.49	0.84	0.58	1.50	0.85
TL ^a^	0.93	0.57	0.76	0.93	0.57	0.76	0.93	0.56	0.38	0.77	0.93	0.56	0.38	0.77	-	-	-	-	-	-	-	-	-	-
TD	0.16	0.09	0.29	0.09	0.12	0.24	0.10	0.11	0.11	0.24	0.10	0.11	0.10	0.24	-	-	-	-	-	-	-	-	-	-
NL ^a^	3.17	2.89	1.93	3.17	2.89	1.93	3.25	2.80	0.69	1.95	3.25	2.80	0.69	1.95	-	-	-	-	-	-	-	-	-	-
ME	0.08	0.13	0.33	0.09	0.13	0.33	0.09	0.13	0.14	0.34	0.09	0.13	0.14	0.34	0.90	1.24	0.90	1.25	0.78	0.58	1.61	0.77	0.59	1.57
TEM	0.07	0.19	0.35	0.07	0.19	0.35	0.06	0.19	0.12	0.35	0.06	0.19	0.12	0.35	0.66	1.70	0.65	1.72	0.52	1.02	1.21	0.52	1.03	1.23

STA: stature; HG: heart girth; BL: body length; RL: rump length; PW: pin width; HW: hook width; RA: rump angle; FA: foot angle; LSV: rear legs–side view; LRV: rear legs–rear view; FUA: fore udder attachment; RUW: rear udder width; UD: udder depth; TL: teat length; TD: teat diameter; NL: navel length; ME: milking ease; TEM: milking temperament. M1: model 1; M2: model 2; M3: model 3; M4: model 4. M1 and M2 assumed CG as a fixed effect, while M3 and M4 assumed CG as a random effect; M1 and M3 fitted only significant fixed environmental effects (*p* < 0.05) in the models, while M2 and M4 fitted all recorded fixed environmental effects. †: traits evaluated under a linear model; ‡: traits evaluated under a threshold model. ^a^: for these traits, model M1 was the same as M2, and model M3 was the same as model M4. §: for this threshold model, the residual variance was assumed as 3.29 (based on the logit scale); -: traits evaluated using only linear models. Standard errors ranged from 0.00 to 1.84, considering all estimates.

**Table 5:** Heritability ($h^{2}$) and repeatability (r) estimates for all evaluated traits using linear and threshold models.

Trait	M1 †		M2 †		M3 †		M4 †		M1 ‡		M2 ‡		M3 ‡		M4 ‡	
	$h^{2}$	r	$h^{2}$	r	$h^{2}$	r	$h^{2}$	r	$h^{2}$	r	$h^{2}$	r	$h^{2}$	r	$h^{2}$	r
STA	0.50	0.79	0.50	0.79	0.43	0.67	0.43	0.67	-	-	-	-	-	-	-	-
HG ^a^	0.26	0.64	0.26	0.64	0.20	0.45	0.20	0.45	-	-	-	-	-	-	-	-
BL	0.29	0.40	0.20	0.38	0.18	0.31	0.18	0.31	-	-	-	-	-	-	-	-
RL ^a^	0.22	0.52	0.22	0.52	0.17	0.35	0.17	0.35	-	-	-	-	-	-	-	-
PW	0.21	0.43	0.21	0.43	0.12	0.24	0.13	0.24	-	-	-	-	-	-	-	-
HW	0.17	0.35	0.19	0.45	0.08	0.16	0.13	0.29	-	-	-	-	-	-	-	-
RA	0.12	0.41	0.12	0.41	0.09	0.33	0.09	0.33	-	-	-	-	-	-	-	-
FA	0.07	0.21	0.07	0.22	0.05	0.16	0.06	0.18	0.07	0.31	0.07	0.33	0.05	0.16	0.06	0.19
LSV	0.09	0.37	0.10	0.37	0.08	0.34	0.08	0.33	0.09	0.40	0.11	0.49	0.08	0.35	0.08	0.35
LRV	0.001	0.37	0.007	0.38	0.006	0.36	0.008	0.36	0.00	0.35	0.01	0.44	0.01	0.31	0.01	0.31
FUA	0.06	0.56	0.07	0.56	0.04	0.38	0.06	0.48	0.06	0.65	0.07	0.66	0.04	0.38	0.05	0.49
RUW	0.07	0.38	0.08	0.37	0.03	0.19	0.06	0.30	0.07	0.52	0.08	0.53	0.09	0.09	0.05	0.28
UD	0.11	0.35	0.11	0.35	0.09	0.30	0.09	0.30	0.12	0.54	0.12	0.54	0.09	0.33	0.09	0.33
TL ^a^	0.41	0.66	0.41	0.66	0.35	0.56	0.35	0.56	-	-	-	-	-	-	-	-
TD	0.30	0.47	0.21	0.47	0.18	0.38	0.17	0.38	-	-	-	-	-	-	-	-
NL ^a^	0.40	0.76	0.40	0.76	0.37	0.70	0.37	0.70	-	-	-	-	-	-	-	-
ME	0.15	0.40	0.15	0.40	0.13	0.31	0.13	0.32	0.17	0.39	0.17	0.40	0.12	0.22	0.12	0.22
TEM	0.11	0.43	0.11	0.42	0.09	0.35	0.09	0.35	0.12	0.42	0.12	0.42	0.09	0.25	0.09	0.25

STA: stature; HG: heart girth; BL: body length; RL: rump length; PW: pin width; HW: hook width; RA: rump angle; FA: foot angle; LSV: rear legs–side view; LRV: rear legs–rear view; FUA: fore udder attachment; RUW: rear udder width; UD: udder depth; TL: teat length; TD: teat diameter; NL: navel length; ME: milking ease; TEM: milking temperament. M1: model 1; M2: model 2; M3: model 3; M4: model 4. M1 and M2 assumed CG as a fixed effect, while M3 and M4 assumed CG as a random effect; M1 and M3 fitted only significant fixed environmental effects (*p* < 0.05) in the models, while M2 and M4 fitted all recorded fixed environmental effects. †: traits evaluated under a linear model; ‡: trait evaluated under a threshold model. ^a^: for these traits, model M1 was the same as M2, and model M3 was the same as model M4. -: traits evaluated using only linear models. Standard errors ranged from 0.01 to 0.05, considering all estimates.

**Table 6:** Adjusted R-squared calculated for all evaluated traits using linear models.

Trait	M1	M2	M3	M4
Stature	0.942	0.942	0.945	0.945
Hearth girth ^a^	0.894	0.894	0.901	0.901
Body length	0.714	0.734	0.746	0.746
Rump length ^a^	0.917	0.917	0.917	0.917
Pin width	0.830	0.830	0.833	0.833
Hook width	0.812	0.865	0.824	0.870
Rump angle	0.828	0.828	0.837	0.837
Foot angle	0.511	0.542	0.555	0.557
Rear legs–side view	0.744	0.729	0.765	0.764
Rear legs–rear view	0.766	0.753	0.796	0.796
Fore udder attachment	0.883	0.886	0.898	0.891
Rear udder width	0.807	0.808	0.832	0.827
Udder depth	0.717	0.717	0.743	0.743
Teat length ^a^	0.901	0.901	0.905	0.905
Teat diameter	0.789	0.815	0.823	0.821
Navel length ^a^	0.951	0.951	0.954	0.954
Milking ease	0.748	0.748	0.764	0.764
Milking temperament	0.755	0.755	0.775	0.775

M1: model 1; M2: model 2; M3: model 3; M4: model 4. M1 and M2 assumed CG as a fixed effect, while M3 and M4 assumed CG as a random effect; M1 and M3 fitted only significant fixed environmental effects (*p* < 0.05) in the models, while M2 and M4 fitted all recorded fixed environmental effects. ^a^: for these traits, model M1 was the same as M2, and model M3 was the same as model M4.

**Table 7:** Average estimated breeding value accuracies for bulls with at least 20 daughters with records for all evaluated traits using linear and threshold models.

Trait	N	M1 †	M2 †	M3 †	M4 †	M1 ‡	M2 ‡	M3 ‡	M4 ‡
STA	53	0.931	0.931	0.935	0.935	-	-	-	-
HG ^a^	52	0.883	0.883	0.896	0.896	-	-	-	-
BL	52	0.912	0.865	0.879	0.879	-	-	-	-
RL ^a^	53	0.872	0.872	0.885	0.885	-	-	-	-
PW	52	0.871	0.871	0.873	0.874	-	-	-	-
HW	52	0.855	0.858	0.860	0.871	-	-	-	-
RA	32	0.815	0.815	0.818	0.817	-	-	-	-
FA	49	0.743	0.747	0.753	0.753	0.725	0.728	0.743	0.744
LSV	32	0.805	0.799	0.795	0.795	0.802	0.801	0.779	0.795
LRV	32	0.196	0.385	0.437	0.454	0.012	0.372	0.361	0.380
FUA	29	0.741	0.760	0.738	0.754	0.729	0.748	0.724	0.739
RUW	31	0.758	0.776	0.756	0.775	0.751	0.764	0.882	0.757
UD	31	0.819	0.819	0.819	0.819	0.816	0.816	0.818	0.818
TL ^a^	53	0.920	0.920	0.923	0.923	-	-	-	-
TD	53	0.910	0.867	0.878	0.877	-	-	-	-
NL ^a^	30	0.920	0.920	0.927	0.927	-	-	-	-
ME	52	0.841	0.841	0.847	0.848	0.839	0.838	0.850	0.849
TEM	54	0.802	0.801	0.800	0.799	0.798	0.796	0.799	0.798

STA: stature; HG: heart girth; BL: body length; RL: rump length; PW: pin width; HW: hook width; RA: rump angle; FA: foot angle; LSV: rear legs–side view; LRV: rear legs–rear view; FUA: fore udder attachment; RUW: rear udder width; UD: udder depth; TL: teat length; TD: teat diameter; NL: navel length; ME: milking ease; TEM: milking temperament. M1: model 1; M2: model 2; M3: model 3; M4: model 4. M1 and M2 assumed CG as a fixed effect, while M3 and M4 assumed CG as a random effect; M1 and M3 fitted only significant fixed environmental effects (*p* < 0.05) in the models, while M2 and M4 fitted all recorded fixed environmental effects. N: number of bulls with at least 20 daughters with records for each trait. †: traits evaluated under a linear model; ‡: traits evaluated under a threshold model. ^a^: for these traits model M1 was the same as M2 and model M3 was the same as model M4. -: traits evaluated using only linear models.

**Table 8:** Spearman’s rank correlation among estimated breeding values for bulls with at least 20 daughters with records for all evaluated traits between models.

Trait	M1 †M2 †	M1 †M3 †	M2 †M4 †	M3 †M3 ‡	M4 †M4 ‡
STA	1.000	0.991	0.991	-	-
HG	§	0.992	0.992	-	-
BL	0.879	0.922	0.982	-	-
RL	§	0.980	0.980	-	-
PW	0.999	0.978	0.974	-	-
HW	0.991	0.990	0.983	-	-
RA	1.000	0.984	0.987	-	-
FA	0.997	0.990	0.986	0.997	0.996
LSV	0.914	0.938	0.985	0.996	0.996
LRV	0.678	0.791	0.955	0.982	0.974
FUA	0.995	0.989	0.980	0.999	0.997
RUW	0.979	0.996	0.988	0.944	0.995
UD	1.000	0.973	0.975	0.994	0.993
TL	§	0.988	0.988	-	-
TD	0.888	0.924	0.991	-	-
NL	§	0.990	0.990	-	-
ME	1.000	0.985	0.987	0.988	0.988
TEM	0.999	0.992	0.989	0.984	0.984

STA: stature; HG: heart girth; BL: body length; RL: rump length; PW: pin width; HW: hook width; RA: rump angle; FA: foot angle; LSV: rear legs–side view; LRV: rear legs–rear view; FUA: fore udder attachment; RUW: rear udder width; UD: udder depth; TL: teat length; TD: teat diameter; NL: navel length; ME: milking ease; TEM: milking temperament. M1: model 1; M2: model 2; M3: model 3; M4: model 4. M1 and M2 assumed CG as a fixed effect, while M3 and M4 assumed CG as a random effect; M1 and M3 fitted only significant fixed environmental effects (*p* < 0.05) in the models, while M2 and M4 fitted all recorded fixed environmental effects. †: traits evaluated under a linear model; ‡: traits evaluated under a threshold model. -: traits evaluated using only linear models; §: the compared models were the same for the trait.

## Data Availability

The data supporting the findings of this study were provided by the Brazilian Agricultural Research Corporation (EMBRAPA), which is responsible for the technical coordination of the Brazilian National Dairy Gir Breeding Program (PNMGL). Records were obtained from the Brazilian Association of Dairy Gir Breeders (ABCGIL) and the Brazilian Association of Zebu Breeders (ABCZ). The availability of these data is restricted, as they were used under a material transfer agreement. The data supporting the findings of this study are available upon reasonable request by contacting the corresponding author, Dr. João Cláudio do Carmo Panetto (joao.panetto@embrapa.br).

## Supplementary Materials

The following supporting information can be downloaded at: https://www.mdpi.com/article/10.3390/ani16030363/s1, Table S1. Standard errors of the estimated additive genetic, permanent environmental, contemporary groups, and residual variances for all evaluated traits using linear and threshold models. Table S2. Standard errors of the heritability (se$h^{2}$) and repeatability (ser) estimates for all evaluated traits using linear and threshold models. Table S3. Paired student’s *t*-test among average estimated breeding value accuracy estimated for bulls with at least 20 daughters with records for different models within the same trait.

## Abbreviations

The following abbreviations are used in this manuscript:

CG	Contemporary group
$R_{adj}^{2}$	Adjusted R-squared
BLUP	Best linear unbiased prediction
STA	Stature
HG	Heart girth
BL	Body length
NL	Navel length
RL	Rump length
PW	Pin width
HW	Hook width
TL	Teat length
TD	Teat diameter
FA	Foot angle
LSV	Rear legs–side view
LRV	Rear legs–rear view
FUA	Fore udder attachment
RUW	Rear udder width
UD	Udder depth
ME	Milking ease
TEM	Milking temperament
RA	Rump angle
M1	Model 1
M2	Model 2
M3	Model 3
M4	Model 4
GLM	General linear model
H	Herd
YE	Year of evaluation
S	Season of evaluation
E	Conformation trait evaluator
D	Diet
I	Age of the dam as covariate fitted as a linear effect
Q	Age of the dam as covariate fitted as a quadratic effect
AIREML	Average information restricted maximum likelihood
$h^{2}$	Heritability
r	Repeatability
$\sigma_{cg}^{2}$	Contemporary group variance
EBV	Estimated breeding value
FAPESP	Sao Paulo Research Foundation
CAPES	Coordination for the Improvement of Higher Education Personnel
CNPq	National Council for Scientific and Technological Development
EMBRAPA	Brazilian Agricultural Research Corporation
PNMGL	Brazilian National Dairy Gir Breeding Program
ABCGIL	Brazilian Association of Dairy Gir Breeders
ABCZ	Brazilian Association of Zebu Breeders
