# Supplementary material for: Optimized Animal Models for the Genetic Evaluation of Conformation Traits, Milking Ease, and Milking Temperament in Dairy Gir Cattle
Source: Animals (Basel). 2026 Jan 23;16(3):363. doi: 10.3390/ani16030363 (PMC12897387; doi:10.3390/ani16030363)
Supplement: Supplementary file 1 [file animals-16-00363-s001.zip › animals-4051209-supplementary.pdf]

Supplementary Material

Table S1. Standard errors of the estimated additive genetic, permanent environmental, contemporary groups, and residual variances for all evaluated traits using linear and threshold models

Trait	M1†			M2†			M3†				M4†				M1‡		M2‡		M3‡			M4‡		
	**seA**	**sePE**	**seE**	**seA**	**sePE**	**seE**	**seA**	**sePE**	**seCG**	**seE**	**seA**	**sePE**	**seCG**	**seE**	**seA**	**sePE**	**seA**	**sePE**	**seA**	**sePE**	**seCG**	**seA**	**sePE**	**seCG**
**STA**	0.72	0.49	0.10	0.72	0.49	0.10	0.73	0.49	0.31	0.10	0.72	0.49	0.31	0.10	-	-	-	-	-	-	-	-	-	-
**HG**[a]	1.66	1.36	0.47	1.66	1.36	0.47	1.74	1.38	1.84	0.47	1.74	1.38	1.84	0.47	-	-	-	-	-	-	-	-	-	-
**BL**	0.78	0.63	0.38	0.68	0.60	0.36	0.69	0.60	0.46	0.36	0.69	0.60	0.46	0.36	-	-	-	-	-	-	-	-	-	-
**RL**[a]	0.17	0.14	0.07	0.17	0.14	0.07	0.17	0.14	0.22	0.07	0.17	0.14	0.22	0.07	-	-	-	-	-	-	-	-	-	-
**PW**	0.10	0.09	0.05	0.10	0.09	0.05	0.10	0.08	0.18	0.05	0.10	0.08	0.18	0.05	-	-	-	-	-	-	-	-	-	-
**HW**	0.21	0.20	0.13	0.21	1.20	0.11	0.21	0.20	0.66	0.13	0.23	0.20	0.34	0.11	-	-	-	-	-	-	-	-	-	-
**RA**	0.94	1.14	0.85	0.94	1.14	0.85	0.91	1.12	0.78	0.85	0.91	1.12	0.78	0.85	-	-	-	-	-	-	-	-	-	-
**FA**	0.01	0.02	0.01	0.01	0.02	0.01	0.01	0.02	0.02	0.01	0.01	0.02	0.01	0.01	0.09	0.10	0.09	0.11	0.07	0.08	0.10	0.08	0.08	0.07
**LSV**	0.02	0.02	0.02	0.02	0.03	0.02	0.02	0.03	0.01	0.02	0.02	0.03	0.008	0.02	0.13	0.13	0.20	0.20	0.15	0.15	0.07	0.15	0.15	0.08
**LRV**	0.00	0.02	0.02	0.01	0.02	0.02	0.01	0.02	0.01	0.02	0.01	0.02	0.01	0.02	0.00	0.09	0.06	0.14	0.05	0.11	0.06	0.05	0.11	0.06
**FAU**	0.01	0.02	0.02	0.02	0.02	0.02	0.01	0.02	0.02	0.02	0.01	0.02	0.01	0.02	0.22	0.27	0.24	0.28	0.15	0.02	0.26	0.16	0.20	0.12
**RUW**	0.01	0.02	0.01	0.01	0.02	0.01	0.01	0.02	0.04	0.01	0.01	0.02	0.01	0.01	0.17	0.19	0.19	0.21	0.09	0.00	0.47	0.13	0.14	0.21
**UD**	0.02	0.02	0.02	0.02	0.02	0.02	0.02	0.02	0.01	0.02	0.02	0.02	0.01	0.02	0.21	0.20	0.21	0.20	0.14	0.14	0.10	0.14	0.14	0.10
**TL**[a]	0.09	0.06	0.02	0.09	0.06	0.02	0.09	0.06	0.04	0.02	0.09	0.06	0.04	0.02	-	-	-	-	-	-	-	-	-	-
**TD**	0.02	0.01	0.01	0.01	0.01	0.01	0.01	0.01	0.01	0.01	0.01	0.01	0.01	0.01	-	-	-	-	-	-	-	-	-	-
**NL**[a]	0.42	0.32	0.11	0.42	0.32	0.11	0.42	0.31	0.09	0.10	0.42	0.31	0.10	0.10	-	-	-	-	-	-	-	-	-	-
**ME**	0.01	0.01	0.01	0.01	0.01	0.01	0.01	0.01	0.01	0.01	0.02	0.01	0.01	0.01	0.16	0.14	0.16	0.14	0.13	0.11	0.14	0.13	0.11	0.14
**TEM**	0.01	0.02	0.01	0.01	0.02	0.01	0.01	0.02	0.01	0.01	0.01	0.02	0.01	0.01	0.14	0.14	0.14	0.14	0.11	0.11	0.11	0.11	0.11	0.12

STA: stature; **HG**: heart girth; **BL**: body length; **RL**: rump length; **PW**: pin width; **HW**: hook width; **RA**: rump angle; **FA**: foot angle; **LSV**: rear legs – side view; **LRV**: rear legs – rear view; **FUA**: fore udder attachment; **RUW**: rear udder width; **UD**: udder depth; **TL**: teat length; **TD**: teat diameter; **NL**: navel length; **ME**: milking ease; **TEM**: milking temperament.

seA: standard error of additive genetic variance; **sePE**: standard error of permanent environmental variance; **seCG**: standard error of contemporary group; **seE**: standard error of residual variance.

M1: model 1; **M2**: model 2; **M3**: model 3; **M4**: model 4. M1 and M2 assumed CG as a fixed effect, while M3 and M4 assumed CG as a random effect; M1 and M3 fitted only significant fixed environmental effects ($p < 0.05$) in the models, while M2 and M4 fitted all recorded fixed environmental effects.

†: traits evaluated under a linear model; ‡: traits evaluated under a threshold model.

[a]: for these traits, model M1 was the same as M2, and model M3 was the same as model M4.

-: traits evaluated using only linear model.

Table S2. Standard errors of the heritability (seh^2) and repeatability (se$r$) estimates for all evaluated traits using linear and threshold models

Trait	M1†		M2†		M3†		M4†		M1‡		M2‡		M3‡		M4‡	
	seh^2	se$r$	seh^2	se$r$	seh^2	se$r$	seh^2	se$r$	seh^2	se$r$	seh^2	se$r$	seh^2	se$r$	seh^2	se$r$
**STA**	0.03	0.01	0.03	0.01	0.03	0.01	0.03	0.01	-	-	-	-	-	-	-	-
**HG**[a]	0.03	0.01	0.03	0.01	0.02	0.02	0.02	0.02	-	-	-	-	-	-	-	-
**BL**	0.03	0.02	0.03	0.02	0.02	0.02	0.02	0.02	-	-	-	-	-	-	-	-
**RL**[a]	0.03	0.01	0.03	0.01	0.02	0.01	0.02	0.01	-	-	-	-	-	-	-	-
**PW**	0.03	0.02	0.03	0.02	0.02	0.01	0.02	0.01	-	-	-	-	-	-	-	-
**HW**	0.03	0.02	0.03	0.02	0.01	0.01	0.02	0.01	-	-	-	-	-	-	-	-
**RA**	0.03	0.03	0.03	0.03	0.02	0.02	0.02	0.02	-	-	-	-	-	-	-	-
**FA**	0.02	0.02	0.02	0.02	0.01	0.02	0.02	0.02	0.02	0.01	0.02	0.01	0.01	0.01	0.02	0.01
**LSV**	0.02	0.03	0.03	0.03	0.02	0.03	0.02	0.03	0.02	0.01	0.03	0.01	0.02	0.01	0.02	0.01
**LRV**	0.01	0.03	0.01	0.03	0.01	0.03	0.01	0.03	0.00	0.01	0.01	0.01	0.01	0.01	0.01	0.01
**FAU**	0.02	0.03	0.02	0.03	0.02	0.02	0.02	0.02	0.02	0.01	0.02	0.01	0.02	0.01	0.02	0.01
**RUW**	0.02	0.03	0.03	0.03	0.01	0.02	0.02	0.02	0.02	0.01	0.03	0.01	0.01	0.01	0.02	0.01
**UD**	0.03	0.03	0.03	0.03	0.02	0.03	0.02	0.03	0.03	0.01	0.03	0.01	0.02	0.01	0.02	0.01
**TL**[a]	0.03	0.01	0.03	0.01	0.03	0.01	0.03	0.01	-	-	-	-	-	-	-	-
**TD**	0.03	0.02	0.03	0.02	0.02	0.02	0.02	0.02	-	-	-	-	-	-	-	-
**NL**[a]	0.05	0.01	0.05	0.01	0.04	0.02	0.04	0.02	-	-	-	-	-	-	-	-
**ME**	0.03	0.02	0.03	0.02	0.02	0.02	0.02	0.02	0.03	0.01	0.03	0.01	0.02	0.01	0.02	0.01
**TEM**	0.02	0.02	0.02	0.02	0.02	0.02	0.02	0.02	0.02	0.01	0.02	0.01	0.02	0.01	0.02	0.01

STA: stature; **HG**: heart Girth; **BL**: body length; **RL**: rump length; **PW**: pin width; **HW**: hook width; **RA**: rump angle; **FA**: foot angle; **LSV**: rear legs – side view; **LRV**: rear legs – rear view; **FUA**: fore udder attachment; **RUW**: rear udder width; **UD**: udder depth; **TL**: teat length; **TD**: teat diameter; **NL**: navel length; **ME**: milking ease; **TEM**: milking temperament.

M1: model 1; **M2**: model 2; **M3**: model 3; **M4**: model 4. M1 and M2 assumed CG as a fixed effect, while M3 and M4 assumed CG as a random effect; M1 and M3 fitted only significant fixed environmental effects ($p < 0.05$) in the models, while M2 and M4 fitted all recorded fixed environmental effects.

†: traits evaluated under a linear model; ‡: traits evaluated under a threshold model.

[a]: for these traits, model M1 was the same as M2, and model M3 was the same as model M4.

-: traits evaluated using only linear models.

Table S3. Paired student's t-test among average estimated breeding value accuracy estimated for bulls with at least 20 daughters with records for different models within the same trait

Trait	N	M1†M2†	M1†M3†	M2†M4†	M3†M3‡	M4†M4‡
**STA**	53	NS	*	*	-	-
**HG**[a]	52	§	*	*	-	-
**BL**	52	*	*	*	-	-
**RL**[a]	53	§	*	*	-	-
**PW**	52	*	*	*	-	-
**HW**	52	*	*	*	-	-
**RA**	32	*	*	*	-	-
**FA**	49	*	*	*	*	*
**LSV**	32	*	*	*	*	*
**LRV**	32	*	*	*	*	*
**FAU**	29	*	*	*	*	*
**RUW**	31	*	*	*	*	*
**UD**	31	*	NS	NS	*	*
**TL**[a]	53	§	*	*	-	-
**TD**	53	*	*	*	-	-
**NL**[a]	30	§	*	*	-	-
**ME**	52	*	*	*	*	NS
**TEM**	54	*	*	*	NS	*

STA: stature; **HG**: heart girth; **BL**: body length; **RL**: rump length; **PW**: pin width; **HW**: hook width; **RA**: rump angle; **FA**: foot angle; **LSV**: rear legs – side view; **LRV**: rear legs – rear view; **FUA**: fore udder attachment; **RUW**: rear udder width; **UD**: udder depth; **TL**: teat length; **TD**: teat diameter; **NL**: navel length; **ME**: milking ease; **TEM**: milking temperament.

M1: model 1; **M2**: model 2; **M3**: model 3; **M4**: model 4. M1 and M2 assumed CG as a fixed effect, while M3 and M4 assumed CG as a random effect; M1 and M3 fitted only significant fixed environmental effects ($p < 0.05$) in the models, while M2 and M4 fitted all recorded fixed environmental effects.

†: traits evaluated under a linear model; ‡: traits evaluated under a threshold model.

§: the compared models were the same for the trait. **N**: number of bulls with at least 20 daughters with records for each trait.

-: traits evaluated using only linear models.

*: Difference between average accuracies for bulls with more than 20 phenotyped daughters were statistically significant ($p < 0.05$); NS: non-significant.
